# Efficacy of Ayurveda Regimen as an Adjunct to Hydroxyurea in Sickle Cell Disease: Protocol for a Prospective, Randomized, Open-Label, Blinded End Point Exploratory Study

**DOI:** 10.2196/79603

**Published:** 2026-04-29

**Authors:** Danish Javed, Amit Kumar Rai, Sachin Bansal, Babita Yadav, Manisha Shrivastava, Garima Goel, Ashok Kumar, Sanjeev Kumar, Azeem Ahmad, Richa Singhal, Bhagwan S Sharma, Anil Mangal, Bhogavalli Chandrasekhararao, Narayanam Srikanth, Rabinarayan Acharya

**Affiliations:** 1 Department of Ayurveda, Yoga and Naturopathy, Unani, Siddha, and Homoeopathy, All India Institute of Medical Sciences Bhopal, Room no 4, Ground floor, AIIMS Campus Rd, AIIMS Campus, Saket Nagar, Bhopal, Madhya Pradesh, 462020, India, 91 9654978285; 2 Department of Kayachikitsa, Ayurvedic and Unani Tibbia College and Hospital, Government of National Capital Territory of Delhi, New Delhi, India; 3 Department of Medical Oncology and Hematology, All India Institute of Medical Sciences Bhopal, Bhopal, Madhya Pradesh, India; 4 Department of Clinical Research, Central Ayurveda Research Institute, New Delhi, India; 5 Bhopal Memorial Hospital & Research Centre, Bhopal, Madhya Pradesh, India; 6 Department of Pathology and Lab Medicine, All India Institute of Medical Sciences Bhopal, Bhopal, Madhya Pradesh, India; 7 Department of Biochemistry, All India Institute of Medical Sciences Bhopal, Bhopal, Madhya Pradesh, India; 8 Department of Community and Family Medicine, All India Institute of Medical Sciences Bhopal, Bhopal, Madhya Pradesh, India; 9 Department of Ayurveda, Central Council for Research in Ayurvedic Science, Ministry of Ayurveda, Yoga and Naturopathy, Unani, Siddha, and Homoeopathy, Government of India, New Delhi, India; 10 National Institute of Malaria Research, New Delhi, India; 11 Department of Clinical Research, Regional Ayurveda Research Institute, Gwalior, Madhya Pradesh, India

**Keywords:** complementary medicine, *Dadimadi Ghrita*, hydroxyurea, *Punarnavadi Mandura*, sickle cell disease, *Vasaguduchyadi Kwatha*, vaso-occlusive crisis

## Abstract

**Background:**

Sickle cell disease (SCD) is a severe, inherited hemoglobin disorder characterized by chronic hemolysis, vaso-occlusive crises (VOCs), and systemic inflammation. Hydroxyurea is the standard conventional pharmacotherapy for SCD, but it has certain limitations, necessitating the need to explore other safe and effective treatment options for SCD. Ayurveda interventions offer a potential therapeutic approach complementary to conventional medicine for SCD management, with anti-inflammatory, immunomodulatory, and hematopoietic properties.

**Objective:**

This randomized controlled trial will evaluate the efficacy and safety of an Ayurvedic therapeutic regimen as an adjunct to hydroxyurea in SCD management, assessing its impact on hematological parameters, inflammatory biomarkers, VOC frequency, and overall quality of life.

**Methods:**

A PROBE (Prospective, Randomized, Open-Label, Blinded End Point) study will be conducted on individuals of any gender aged 18 years or older and diagnosed with SCD (with hemoglobin S levels more than 60% and a history of at least 1 VOC per year over the past 3 y). Individuals with acute VOC or any severe infection requiring hospitalization, a history of significant comorbidities, or hematopoietic stem cell transplantation will not be considered. The study will be conducted at the All India Institute of Medical Sciences, Bhopal, India. A total of 100 participants will undergo random assignment in a 1:1 ratio to receive either an Ayurveda regimen (*Dadimadi Ghrita*, *Punarnavadi Mandura*, and *Vasaguduchyadi Kwatha*) as an add-on to hydroxyurea or hydroxyurea alone for 8 months. The primary outcome will be a change in hemoglobin electrophoresis parameters (hemoglobin S, fetal hemoglobin, and adult hemoglobin) and the frequency of VOC episodes over 8 months. The secondary outcome measures include changes in the levels of proinflammatory markers (interleukin-6, interleukin-8, C-reactive protein, and transforming growth factor-β) and lactate dehydrogenase, frequency of hospitalization for VOCs and blood transfusions, and health-related quality of life (Short Form-8 Health Survey questionnaire). Safety will be evaluated by recording the incidence of adverse events and changes in liver and kidney function tests from baseline.

**Results:**

The recruitment of study participants was initiated on November 1, 2023. By the second week of February 2025, 83 participants had been enrolled in the study. The final study is expected to be complete by December 31, 2025. We will start the analysis of the study outcomes in February 2026, and the publication of the final results is expected by August 2026.

**Conclusions:**

This randomized controlled trial protocol outlines a rigorous study design aimed to explore the potential benefits of an integrated therapeutic regimen comprising Ayurveda interventions and standard conventional care in the long-term management of SCD through validated clinical and laboratory parameters. The outcomes of this study can address the needs and challenges associated with SCD management and inform future management protocols.

## Introduction

### Background and Rationale

Sickle cell disease (SCD) is one of the most common hereditary disorders globally, particularly in the African, West Mediterranean, Middle Eastern, and Indian subcontinent regions [1,2]. It is an autosomal recessive hemoglobinopathy caused by a point mutation in the β-globin gene (located on chromosome 11), resulting in the production of abnormal hemoglobin S (HbS) [3]. Under hypoxic conditions, polymerization of deoxygenated sickle hemoglobin occurs, leading to erythrocyte deformation, increased cellular rigidity, vaso-occlusion, chronic hemolysis, and endothelial dysfunction [1,2,4]. These pathophysiological mechanisms contribute to severe clinical manifestations, including recurring episodes of vaso-occlusive crises (VOCs); hemolytic anemia; thrombotic complications; aseptic necrosis of bone; microinfarction of spleen, brain, and kidney; stroke; multiorgan damage; increased susceptibility to repeated infections due to a weakened immune system; and ultimately reduced life expectancy [3,5,6].

The global prevalence of SCD was 7.74 million in 2021, with an increase of 41.4% from 2000 [7]. SCD has a strikingly high contribution to the all-cause mortality burden, with 376,000 deaths reported in 2021, ranking 12th across all causes [7]. It is estimated that approximately 300,000 infants are born with SCD annually, with 80% from low- and middle-income countries [5]. India contributes significantly to the global SCD burden and is one of the most affected nations in the prevalence of births with SCD [8]. Madhya Pradesh, Chhattisgarh, and Maharashtra states have a high prevalence of SCD in India, and the burden is higher among the indigenous tribal communities [9,10]. In Madhya Pradesh (a major state in central India), this genetic variant represents a significant public health challenge, with an estimated 961,492 individuals being sickle cell heterozygotes and 67,861 individuals being sickle cell homozygotes [11]. Notably, approximately 27 out of the 55 districts in the state fall within the sickle cell belt, where the prevalence of HbS ranges from 10% to 33% [11].

Hydroxyurea, the primary disease-modifying pharmacotherapy for SCD, has been reported to effectively enhance fetal hemoglobin (HbF) production, decrease erythrocyte adhesion to the endothelium, and reduce VOCs, transfusion requirements, and hospitalizations [12,13]. However, there is an inadequate therapeutic response with HU in preventing chronic complications of SCD [14,15]. There is also insufficient evidence about the long‐term safety, as dose-limiting toxicity with HU has been reported [14,15]. This necessitates the exploration of complementary and adjunctive therapies for safe and effective long-term management of SCD. Several studies have reported the utilization of traditional herbal medicine in SCD management, especially in Africa and India [16-19]. Surveys from Uganda, India, and Nigeria show that traditional medicine (TM) is often combined with conventional care due to its perceived efficacy, affordability, and fewer adverse effects (AEs). Ethnopharmacological investigations have identified several plants with antisickling, antioxidant, and red blood cell–stabilizing properties. Some interventions have demonstrated reductions in pain crises, improvements in hematological parameters, and acceptable safety profiles; however, certain herbal products may interact with conventional medications. Despite this potential, challenges persist, including inconsistent herbal compositions, limited standardization, and a lack of high-quality randomized controlled trials (RCTs). Integrating evidence-based TM with conventional therapy may enhance patient-centered SCD care in resource-limited settings.

Given the increasing global emphasis on TM, Ayurveda has garnered attention for its potential role in the safe and effective management of various hematological disorders, including SCD.

The clinical presentation of *Tridoshaja* or *Sannipatika Pandu Roga* described in Ayurveda closely resembles chronic hematological disorders, including SCD [20]. Furthermore, the hereditary origin of SCD is also described in Ayurveda as *Sahaja Vikara* (inherited disorders) [20]. Ayurveda interventions indicated for the management of hematological disorders, such as *Punarnavadi Mandura*, *Dadimadi Ghrita*, and *Vasaguduchyadi Kwatha*, have demonstrated hematinic, anti-inflammatory, antioxidant, adaptogenic, immunomodulatory, analgesic, antimicrobial, antipyretic, and hepatoprotective properties [21-29]. Few case reports, observational studies, and exploratory clinical studies have reported promising outcomes of Ayurveda interventions in SCD management in terms of relief in clinical features, reduced frequency of VOCs, improved hematological parameters, reduced need for blood transfusion and hospitalization, better quality of life, and good tolerability and safety [30-34]. Furthermore, some experimental studies have also reported the antisickling activity of a few herbal interventions [20,35,36]. A recent scoping review has reported promising outcomes of herbal and ayurvedic management in SCD [37]. The available evidence suggests that Ayurveda formulations may enhance hemoglobin levels, modulate inflammatory cytokines, reduce oxidative stress, and improve microvascular circulation, potentially mitigating disease severity in SCD. Despite these promising observations, well-designed RCTs evaluating the efficacy and safety of Ayurveda interventions in SCD remain scarce. Therefore, robust RCTs and pharmacokinetic evaluations are needed to ensure the safety, efficacy, and reliable use of Ayurveda interventions.

Considering this limitation, this study is conceptualized to rigorously evaluate the therapeutic efficacy and safety of an Ayurvedic regimen in combination with hydroxyurea in individuals with SCD. The study will employ robust clinical methodologies, including comprehensive hematological assessments (hemoglobin electrophoresis, reticulocyte count, and red cell indices), inflammatory biomarkers (interleukin-6 [IL-6], interleukin-8 [IL-8], C-reactive protein [CRP], and transforming growth factor-β [TGF-β]), and quality of life measures using validated patient-reported outcome instruments. Given the high disease burden of SCD in India and worldwide and the increasing scientific recognition of integrative medicine, this research has the potential to shape future treatment paradigms for SCD. The findings will contribute critical data on the feasibility and clinical impact of combining traditional and contemporary medical approaches, ultimately advancing the standard of care and improving outcomes for individuals affected by SCD.

### Objectives

The present RCT is designed to assess the therapeutic efficacy of the Ayurveda regimen as an adjunct to hydroxyurea in terms of change in hemoglobin electrophoresis, complete blood count (CBC), and episodes of painful crises (VOCs) in individuals with SCD compared to hydroxyurea alone. The key secondary objectives of this study include evaluating the efficacy of the proposed integrated regimen for SCD on clinical parameters, proinflammatory biomarkers, need for blood transfusion and hospitalization, and quality of life parameters. Another secondary objective is to assess the safety of the proposed therapeutic regimen in managing SCD.

### Study Hypotheses

#### Primary Hypotheses

(H1) The Ayurveda regimen added to hydroxyurea will reduce the frequency of VOCs over 8 months compared with hydroxyurea alone (targeted detectable relative reduction ≥30%). (H2) The Ayurveda + hydroxyurea group will demonstrate a favorable change in hemoglobin electrophoresis parameters (reduction in %HbS and increase in %HbF) versus hydroxyurea alone at 8 months.

#### Secondary Hypotheses

The Ayurveda +hydroxyurea group will demonstrate (1) reductions in proinflammatory biomarkers (IL-6, IL-8, CRP, and TGF-β) and lactate dehydrogenase (LDH); (2) fewer hospitalizations and blood transfusions; (3) improvement in patient-reported outcomes, including pain (Brief Pain Inventory [BPI]), fatigue (Modified Fatigue Impact Scale [MFIS]), and health-related quality of life (HRQoL; assessed through SF-8 [Short Form-8 Health Survey]); and (4) a safety profile comparable to HU alone as evaluated by the incidence of AEs and liver function test (LFT)/kidney function test (KFT) changes.

## Methods

This study protocol has been designed in accordance with the SPIRIT (Standard Protocol Items: Recommendations for Interventional Trials) guidelines (Checklist 1).

### Study Design and Setting

This PROBE (Prospective, Randomized, Open-Label, Blinded End Point) clinical study will be conducted at the All India Institute of Medical Sciences (AIIMS), Bhopal, a tertiary care center in Madhya Pradesh, India. This region has a high prevalence of SCD. The study participants will be recruited from the Hematology Outpatient Department at AIIMS, Bhopal. The trial duration will span 30 months, including participant recruitment, administration of study interventions, follow-up assessments, and data analysis.

### Study Participants

Individuals of any gender aged 18 years or above diagnosed with SCD (based on hemoglobin electrophoresis or high-performance liquid chromatography) with HbS levels more than 60%, not on hydroxyurea for at least 4 months before enrollment, a history of at least 1 VOC per year over the past 3 years, hemoglobin above 5 g/dL, platelet count more than 100,000/mcL, international normalized ratio ≤2.0, and partial thromboplastin time ≤48 seconds will be included in the study.

Individuals with acute VOC, uncontrolled intercurrent illness, or any severe infection requiring hospitalization; history of frequent hospitalizations (≥10 times) for SCD-related painful crises in the last 12 months; significant comorbid conditions, such as coronary artery disease, uncontrolled hypertension (more than 160/100 mm Hg) even after medications; diabetes mellitus; severe chronic obstructive pulmonary disease; malignancy; HIV infection; any neurological disorder or psychiatric illness; bleeding diathesis; impaired renal function (serum creatinine more than 1.5 mg/dl) and abnormal hepatic function (alanine aminotransferase more than 3 times the upper limit of normal); a history of recent blood transfusions within the last 1 month; anticoagulation or antiplatelet therapy; and prior hematopoietic stem cell transplantation or solid organ transplant will be excluded from the study. Similarly, those with a history of hypersensitivity to the study interventions, pregnant or lactating women, or those with any other clinical condition that the investigator believes may compromise the participant’s safety or compliance or interfere with evaluation will not be considered for the study.

### Study Intervention

The eligible participants in the Ayurveda add-on group will receive a comprehensive Ayurveda regimen (*Dadimadi Ghrita*, *Punarnavadi Mandura*, and *Vasaguduchyadi Kwatha*) combined with hydroxyurea (10‐15 mg/kg/day) for a period of 8 months. The details of the Ayurveda interventions are provided in Table 1. The participants in the control group will be advised to take only hydroxyurea (10‐15 mg/kg/day) for 8 months.

**Table 1.** Details of interventions in the Ayurveda regimen.

Intervention	Dosage with frequency	*Anupana* (vehicle of administration)	Duration, days
*Dadimadi Ghrita* (API^a^ Part II, Vol II)	12 gm twice daily before food	Lukewarm water	240
*Punarnavadi Mandura* (API Part II, Vol III)	500 mg twice daily after food	Lukewarm water	240
*Vasaguduchyadi Kwatha* (AFI^b^ Part I)	40 ml twice daily after food	—^c^	240

a API: Ayurvedic Pharmacopoeia of India.

b AFI: Ayurvedic Formulary of India.

c Not applicable.

Ayurveda practitioners in the research team will administer the Ayurveda interventions at the study sites throughout the trial period. The quality standards and dosage of the study interventions complied with those outlined in the Ayurvedic Pharmacopeia of India. The trial Ayurveda interventions are procured from Indian Medicines Pharmaceutical Corporation Limited, Ministry of Ayush, Government of India.

### Discontinuation of the Study Interventions

If any participant develops any AEs, such as gastrointestinal symptoms, any allergic response, or a change in biochemical parameters, the administration of the trial interventions will be temporarily stopped, and the participant will be closely monitored. If symptoms recur after reintroducing the study interventions, the participant will be withdrawn from the study after assessing the causality of the AE or the adverse drug reaction. All such events will be recorded in the AE or adverse drug reaction reporting format.

### Compliance With Trial Interventions During the Study

All the study participants will be provided with an information leaflet containing instructions for the use (dose, frequency, and time of administration) and storage of the study interventions. The participants will also be issued a compliance form during the baseline and subsequent follow-up visits to self-report their consistent or irregular use of trial interventions and to record any missed doses with remarks for missing, which will enable the assessment of adherence to the dosing pattern as per the study protocol. During each follow-up visit, the participants will be asked to return the used/unused/partially used containers of the study interventions to the investigators to assess adherence and cross-check with the participant self-reported compliance form.

The participants who do not adhere to the study protocol, do not have 80% or more compliance, develop any study-related AEs, or withdraw their voluntary consent for participation in the study will be withdrawn from the study.

### Concomitant or Rescue Medication

The investigators would monitor the participants for any concomitant or rescue medication they required during the study period. All instances of concomitant care would be carefully documented in the case record form (CRF). If any participant develops a serious AE or treatment-emergent AE during the study period, the participant would be withdrawn and given appropriate incidental care at the AIIMS, if needed. The sponsor and the Institutional Ethics Committee will be notified about the same within 2 working days, along with appropriate justification.

### Outcome Measures

The primary outcome is the change in the levels of hemoglobin electrophoresis parameters (HbS, HbF, and HbA), CBC parameters, frequency of painful crisis (VOCs), and change in the BPI score from baseline. The secondary outcome measures include the change in the levels of proinflammatory markers, such as IL-6, IL-8, CRP, TGF-β, and LDH; change in the clinical parameters, such as general weakness, myalgia, headache, backache, abdominal pain, anorexia, and palpitation (assessed through the VAS [Visual Analog Scale] score); change in the score of MFIS; time duration (in d/mo) for the first VOC after the administration of trial interventions; frequency of hospitalization for VOCs and blood transfusions; and change in the HRQoL (assessed through the SF-8 questionnaire).

The outcomes will be evaluated every 15 days for 4 months, followed by every 30 days for the next 4 months (ie, until the completion of 8 mo from baseline). Hemoglobin electrophoresis and serum proinflammatory markers will be evaluated at baseline, day 60, day 120, day 180, and day 240.

### Outcome Assessment and Laboratory Methods

All assessments are performed by trained study personnel according to standard operating procedures. The primary and secondary outcomes will be measured as follows:

- Hemoglobin electrophoresis (HbS, HbF, and HbA): quantified by high-performance liquid chromatography/capillary electrophoresis (a standardized method used at the NABL [National Accreditation Board for Testing and Calibration Laboratories]-accredited laboratory at the study site). The results will be reported as a percentage of total hemoglobin [38,39].
- CBC and red cell indices: measured using an automated hematology analyzer at the NABL-accredited laboratory at the study site; reticulocyte count will be reported using the automated or manual method as available.
- LDH: serum LDH measured using the standard enzymatic assay at the laboratory.
- CRP: measured by immunoturbidimetric assay.
- Cytokines (IL-6, IL-8, and TGF-β): quantified using commercially available sandwich ELISA (enzyme-linked immunosorbent assay) kits according to the manufacturer’s instructions; kit sources and detection ranges will be recorded in the laboratory log [40,41].
- Pain and symptom severity: pain intensity and symptom-specific severity (generalized weakness, myalgia, headache, backache, abdominal pain, anorexia, and palpitation) will be recorded using a 0 to 10 VAS and the BPI for pain impact [42,43].
- Fatigue: assessed using the MFIS [44].
- HRQoL: assessed using the SF-8 questionnaire; the validated Hindi version will be used for Hindi-speaking participants. Scoring follows standard SF-8 scoring algorithms [45,46].
- VOC and disease events: VOC is defined as an acute episode of pain requiring analgesic therapy and medical attention. The number of VOCs, time of first VOC after randomization (in days), VOC-related hospitalizations, and the number of blood transfusions will be recorded from participant diaries and verified using hospital records.
- Adherence/drug compliance: compliance with Ayurveda formulations and HU will be evaluated by (1) the participant daily compliance diary, (2) pill/container return with pill count or weight where applicable, and (3) participant self-reports at each visit. Participants with less than 80% overall adherence (as determined by pill count or diary) will be considered nonadherent per protocol.

Time points for each assessment are described in Table 2 (baseline, days 60, 120, 180, and 240 for key biomarkers).

**Table 2.** Study schedule.

Screening	Baseline	Treatment (mo)							
		1	2	3	4	5	6	7	8
Informed consent	X^a^	—^b^	—	—	—	—	—	—	—
Demographics	X	—	—	—	—	—	—	—	—
Medical history	X	—	—	—	—	—	—	—	—
Review of sickle cell disease–related earlier investigations, if any	X	—	—	—	—	—	—	—	—
Vitals	X	XX^c^	XX	XX	XX	X	X	X	X
Physical examination	X	XX	XX	XX	XX	X	X	X	X
Clinical assessment	X	XX	XX	XX	XX	X	X	X	X
Quality of life	X	XX	XX	XX	XX	X	X	X	X
CBC^d^	X	XX	XX	XX	XX	X	X	X	X
LFT^e^	X	XX	XX	XX	XX	X	X	X	X
KFT^f^	X	XX	XX	XX	XX	X	X	X	X
LDH^g^	X	XX	XX	XX	XX	X	X	X	X
Urine analysis	X	XX	XX	XX	XX	X	X	X	X
HbS^h^, HbF^i^, HbA^j^	X	—	X	—	X	—	X	—	X
Inflammatory biomarkers	X	—	X	—	X	—	X	—	X
Pregnancy test	X	—	—	—	—	—	—	—	—
Issue of trial drugs	—	XX	XX	XX	XX	X	X	X	X
Drug compliance	—	XX	XX	XX	XX	X	X	X	X
Disease evaluation	—	XX	XX	XX	XX	X	X	X	X
Assessment of adverse events	—	XX	XX	XX	XX	X	X	X	X
Concomitant medication	—	XX	XX	XX	XX	X	X	X	X
Need of rescue analgesic medication for VOCs^k^	—	XX	XX	XX	XX	X	X	X	X

a X: once a month.

b Not applicable.

c XX: twice a month with a 15-day gap period.

d CBC: complete blood count.

e LFT: liver function test.

f KFT: kidney function test.

g LDH: lactate dehydrogenase.

h HbS: hemoglobin S.

i HbF: fetal hemoglobin.

j HbA: adult hemoglobin.

k VOC: vaso-occlusive crisis.

### Safety Assessment

The safety of the trial interventions will be determined by recording the incidence of AEs, if any, during scheduled follow-up visits in a structured format. All AEs during the study would be recorded and monitored as per International Council for Harmonisation of Technical Requirements for Pharmaceuticals for Human Use (ICH) Good Clinical Practice (GCP) guidelines. Safety would also be evaluated by the assessment of LFT, KFT, and urine routine and microscopic examination conducted every 15 days for 4 months, followed by every 30 days for the next 4 months.

### Handling of Potential Pharmacokinetic and Pharmacodynamic Interactions

While direct human pharmacokinetic data on interactions between hydroxyurea and the selected Ayurvedic formulations are limited, we recognize the possibility of pharmacodynamic and pharmacokinetic interactions (eg, altered hepatic metabolism, modulation of drug transporters, and changes in absorption or renal clearance). To ensure participant safety, the following are considered:

1. Concomitant medications will be recorded at each visit.
2. Routine monitoring of hepatic (LFT) and renal (KFT) parameters is performed frequently (every 15 d for the first 4 mo and monthly thereafter) to detect early biochemical signals.
3. Any clinically significant laboratory changes or suspected interactions will prompt temporary discontinuation and further investigation; the Data and Safety Monitoring Board (DSMB) will review any signals for interaction and recommend further pharmacokinetic/pharmacodynamic substudies if warranted.
4. All serious adverse events and suspected unexpected serious adverse reactions will be reported to the institutional ethics committee and sponsor in accordance with regulatory procedures.

### Sample Size

Based on the results from a previous study comparing hydroxyurea with placebo [47], where the difference in the rate of VOCs between the 2 groups was 44%, this study has been designed to have 80% statistical power to detect a difference of at least 30% in the rate of VOCs between the Ayurveda regimen alongside the hydroxyurea group compared to the hydroxyurea alone group at a 95% CI. To achieve this, 42 participants per group are required. Accounting for an expected attrition rate of 20%, the final sample size for each group is determined to be 50 participants. Therefore, a total of 100 participants will be enrolled in the study.

### Recruitment of Study Participants

During the study, the investigators will screen individuals with clinical features of SCD who are visiting the Hematology Outpatient Department of AIIMS, Bhopal, based on the defined inclusion and exclusion criteria to identify and recruit potential participants. The eligible participants would be allocated to either of the 2 groups based on a computer-generated randomization schedule. The screening process will continue until the target sample size for the study is achieved.

### Randomization

The eligible participants will be randomly assigned to an add-on or a control group with a 1:1 allocation. SPSS version 28.0 (IBM Corp) will be used to generate the random number sequences. An independent statistician, not involved in the participant’s enrollment and assessment, will generate the randomization sequence.

### Allocation Concealment

Sequentially numbered, opaque, sealed envelopes will be used to ensure the allocation concealment. The participant’s enrollment number will be printed on the top of the envelope, and a slip on which the participant’s allocated group is printed will be kept inside the envelope. After completing all baseline assessments, the research staff will provide the sealed envelope to the eligible participants. The participants will open the envelope and then be allocated to the group as per the slip inside the envelope. The opened envelope and the printed slip will be attached to the participant’s CRF for record and trial monitoring.

### Data Collection

The baseline demographics and clinical and physical examination aspects will be collected by qualified study personnel and reported in a CRF designed for the purpose. The subjective and objective outcome assessments will be performed as per the study protocol (Table 2).

The blood sample for the objective assessment parameters, such as hemoglobin electrophoresis, proinflammatory biomarkers, LDH, CBC, LFT, and KFT, would be collected during scheduled follow-up visits and transported to the NABL-accredited laboratory, and the data received from the lab would be entered into the CRF and electronic CRF (e-CRF). The research team will undergo training on the study protocol, standard operating procedure for the conduct of the study, storage and dispensing of study interventions, handling of biological samples, data collection, and recording to ensure compliance with GCP principles while ensuring participant safety, data accuracy, and reliability.

### Data Management

Data management in this clinical study will adhere to stringent guidelines to ensure the accuracy, reliability, and integrity of collected information. Upon the participant’s assessment, the research team would promptly enter the data into CRFs and e-CRFs. Source documents and CRFs will be securely stored in access-restricted areas, limited solely to the study team. e-CRFs would be password-protected and stored in secure, access-restricted computer systems.

Data entered by the study personnel would undergo meticulous cross-verification by the study investigators at the study site, ensuring the reliability of the data. Rigorous quality measures would be implemented, such as regular audits, to identify and address any discrepancies in the data. The data management practices would adhere to regulatory guidelines and ethical principles, prioritizing the protection of participant rights.

### Statistical Methods

#### Analyses

The categorical data will be presented as numbers (percentages) and compared between groups using the *χ*^2^ test. Continuous data following normal distribution will be reported as mean (SD), and between-group comparisons will be performed by the independent sample *t* test. Within-group comparisons for normal data will be carried out by the paired sample *t* test/repeated measures ANOVA. The nonnormal data will be reported as median (IQR), and between-group comparisons will be performed using the Mann-Whitney *U* test. Within-group comparisons will be carried out using the Wilcoxon signed rank test/Friedman test. Kaplan-Meier survival analysis will be conducted for time-to-event outcomes. Regression analysis will be performed to study the effect of confounding variables on the study outcomes. A *P* value less than .05 will be considered significant. SPSS version 28.0 will be used for statistical analysis.

Both intention-to-treat and per-protocol populations will be used for efficacy analysis. The modified intention-to-treat (mITT) analysis approach will be applied to handle missing data. The missing data of all those participants for whom data are available for at least 1 visit post baseline (day 30) will be imputed. The last observation carried forward method will be used for imputing the missing values. The population for safety analysis will include all participants who receive at least a single dose of the trial interventions, ensuring a comprehensive safety assessment.

#### Overview and Primary Analysis Population

The primary efficacy analysis will be performed on the mITT population, which is defined as all randomized participants who receive at least 1 dose of the study intervention and have at least 1 postbaseline assessment. The secondary analysis will be performed on the PP (participants with ≥80% adherence and no major protocol violations). The safety population will include all participants who receive at least 1 dose of the study intervention.

#### Primary End Points and Tests

The primary end points of the study are designed to evaluate the core clinical outcomes associated with the intervention.

- VOC frequency (count data over 8 mo): We will compare event rates between groups using Poisson regression or, if overdispersion is present, negative-binomial regression with exposure time as an offset. Rate ratios with 95% CIs will be reported.
- Change in hemoglobin electrophoresis parameters (%HbS and %HbF): Between-group differences in mean change from baseline to 8 months will be assessed using independent-sample *t* tests if data are approximately normal; otherwise, Mann-Whitney *U* test. The repeated measures of these parameters across time points will be analyzed using repeated measures ANOVA or mixed-effects linear models (participant as random effect) to account for within-subject correlation.

#### Secondary End Points and Tests

The secondary end points are included to explore additional effects of the intervention and provide supportive evidence to the primary findings.

- Continuous biomarkers (IL-6, IL-8, TGF-β, CRP, LDH, and CBC indices): Between-group comparisons of change from baseline will use *t* tests or nonparametric equivalents; longitudinal analyses will use mixed-effects models. Biomarkers with skewed distributions will be log-transformed as appropriate.
- Time of first VOC: Kaplan-Meier survival curves and log-rank test; hazard ratios were estimated using Cox proportional hazards models adjusting for baseline covariates.
- Hospitalization and transfusion counts: analyzed similarly to VOC counts (Poisson/negative binomial).
- Patient-reported outcomes (BPI, MFIS, and SF-8): between-group comparisons were conducted using *t* tests or mixed models for repeated measures.

#### Adjustment for Covariates and Multiplicity

Primary analyses will adjust for prespecified baseline covariates known to influence SCD outcomes (age, sex, baseline HbF, and baseline VOC rate). For multiple secondary outcomes, *P* values will be interpreted cautiously; where appropriate, we will report false discovery rate–adjusted *P* values or Bonferroni correction for prespecified families of end points.

#### Missing Data and Sensitivity Analyses

Primary analyses will use the last observation carried forward for the primary mITT approach (consistent with the planned protocol). We will also perform sensitivity analyses using multiple imputation under missing-at-random assumptions, and worst-case/best-case imputation to assess robustness under different missingness mechanisms. PP analyses and analyses excluding participants with major protocol deviations will also be reported. All missing data patterns and reasons for dropout will be summarized in the CONSORT (Consolidated Standards of Reporting Trials) flow diagram and a missingness table.

#### Regression and Confounding

Multivariable regression models will be used to explore associations and adjust for potential confounders. Interaction terms (eg, baseline HU dose×treatment group) will be explored where clinically relevant.

#### Software and Significance Threshold

Analyses will be conducted using SPSS version 28.0 and R software (R Foundation for Statistical Computing), where required. A 2-sided *P*<.05 will be considered statistically significant unless otherwise specified for multiple comparisons.

### Monitoring

The DSMB will monitor the study for quality and regulatory compliance. The DSMB will review the progress of the study every 6 months till the end of the study period.

### Trial Audit

The onsite monitoring visit by an independent committee constituted by the sponsor will be planned to ensure that the study procedures and data collection processes comply with the existing regulatory standards and to check the accuracy, completeness, legibility, and timeliness of the reported study data.

### Ethical Considerations

The Institutional Ethics Committee of AIIMS, Bhopal, has approved the study protocol and related documents (vide letter number IHEC-LOP/2022/EL064 dated March 6, 2023) to ensure compliance with ethical standards and safeguard the rights and well-being of the participants. The study has been registered prospectively at the Clinical Trial Registry of India (CTRI/2023/03/051032). The study will be undertaken following the principles of the Declaration of Helsinki, ICMR’s National Ethical Guidelines for Biomedical and Health Research on Human Participants [48] (2017), and ICH GCP guidelines [49]. All substantial amendments in the study protocol affecting patient safety or study integrity will be presented to the institutional ethics committee for approval before implementation in the study. Before undergoing any study-related procedure, the potential participants would receive a participant information sheet in Hindi or their native language. The participant information sheet would comprehensively outline the various aspects of the study, equipping the participants with the necessary information to make an informed decision regarding participation in the study. The written consent will be obtained in the consent form, signed by the participant and the study personnel delegated for the task.

All the relevant study data will be stored securely at the study site with password-protected access systems in areas with limited access. To maintain participant confidentiality, a coded enrollment identification number will be used to identify all laboratory specimens, reports, data collection, and relevant forms. All records containing names or other personal identifiers, such as informed consent forms, will be stored separately from the study records identified by a code identification number in an area with limited access.

### Ancillary and Posttrial Care

No ancillary studies are proposed with the present clinical study. If required, the participants will be provided with routine medical care after completing the study.

## Results

The recruitment of study participants was initiated on November 1, 2023. As of the second week of February 2025, 83 participants had been enrolled in the study. The final study is expected to be completed by December 31, 2025. We will start the analysis of the study outcomes in February 2026, and the publication of the final results is expected by August 2026.

## Discussion

### Expected Findings

SCD remains a global public health burden affecting millions of individuals worldwide. This hereditary disorder is considerably prevalent in the Indian subcontinent and sub-Saharan African regions. The clinical manifestations and complications associated with SCD significantly impact the physical and psychosocial well-being of individuals affected by the disease. Due to unsatisfactory long-term relief and associated AEs with conventional medications, a substantial proportion of individuals affected with SCD prefer TM systems and herbal medicines, especially in endemic regions, such as India and Africa. Furthermore, exploratory clinical studies and case reports on Ayurveda interventions in SCD management also highlighted their safety and beneficial effects, such as clinical improvement, reduced frequency of VOCs, reduced need for blood transfusion and hospitalization, and improved quality of life [30-34]. A few herbal interventions were also reported to have antisickling activity [35,36]. A systematic review also concluded that herbal medicines may have a potentially beneficial effect in reducing VOCs in SCD [50].

Based on the aforesaid preliminary leads, the present RCT is conceptualized to evaluate the efficacy of an Ayurveda regimen (*Punarnavadi Mandura*, *Dadimadi Ghrita*, and *Vasaguduchyadi Kwatha*) as an adjunct to hydroxyurea for the management of SCD. The ingredients of the proposed interventions, such as *Punarnavadi Mandura,* have hematinic, anti-inflammatory, immunomodulatory, antioxidant, hepatoprotective, spasmolytic, and antifibrinolytic activities [51,52]. Furthermore, a case report on Ayurveda interventions, such as *Punarnavadi Mandura* and *Arogyavardhini Vati,* in SCD highlighted that there were no episodes of VOCs during the treatment period, along with significant clinical relief, and a slight decrease in HbS levels [34]. In addition, *Punarnavadi Mandura* and *Dadimadi Ghrita* were reported to have therapeutic efficacy in iron deficiency anemia and showed a significant effect on relevant hematological parameters [21-24]. An experimental study also highlighted that *Punarnavadi Mandura* reversed the pathological changes associated with hemolytic anemia, restored the hematological parameters, downregulated the proinflammatory cytokines, offset the splenomegaly, and positively modulated erythropoiesis [25]. *Vasaguduchyadi Kwatha* is indicated in Ayurveda for different hematological disorders, and its ingredients are reported to have antioxidant, anti-inflammatory, hepatoprotective, immunomodulatory, and hematopoietic activities [26-28].

Therefore, the Ayurveda interventions in the proposed therapeutic regimen have the potential to effectively manage the clinical manifestations and complications due to SCD. Furthermore, the herbal ingredients of the proposed interventions contain anti-inflammatory, antioxidant, immunomodulatory, hematinic, analgesic, antimicrobial, antipyretic, and hepatoprotective properties. Therefore, these interventions could play a role in stabilizing erythrocyte morphology and reducing oxidative stress.

### Mechanistic Framework: Putative Pathways for Hematologic Benefits

The selected Ayurvedic formulations have constituent ingredients and prior experimental/clinical evidence suggesting hematinic, antioxidant, and anti-inflammatory effects, which provide a plausible mechanistic basis for potential benefits in SCD. These agents may (1) reduce oxidative stress on erythrocytes (thereby decreasing hemolysis and hemoglobin degradation), (2) modulate inflammatory cytokines (IL-6, IL-8, and TGF-β) implicated in VOC pathogenesis and endothelial activation, (3) support erythropoiesis via hematinic and hepatoprotective effects, and (4) improve microvascular circulation through anti-inflammatory and antioxidant actions. These combined actions could reduce the rate and severity of VOCs and favorably influence hemoglobin composition (increase effective erythropoiesis and possibly %HbF indirectly), providing a biologically plausible rationale for evaluating these formulations as adjuncts to hydroxyurea.

Given the chronic nature of SCD and associated morbidity, integrating Ayurveda with contemporary pharmacotherapy could offer a novel therapeutic avenue for better clinical outcomes. The Ayurveda regimen, when combined with hydroxyurea, may contribute to a reduction in the frequency and severity of VOCs, a key determinant of disease burden. This integrative approach could revolutionize SCD management, particularly in regions with a high prevalence of the disease and limited access to advanced medical care. Moreover, mechanistic studies exploring the molecular pathways through which these formulations exert their effects will provide deeper insights into their therapeutic potential.

The present RCT protocol has several key strengths. First, it uses polyherbal Ayurveda medications, which are easy to administer, tolerable, and safe, with the potential to address SCD-related pathological changes. Second, the study includes an adequate sample size, validated clinical assessment tools, and relevant biomarkers as outcomes to address various parameters related to the pathogenesis of SCD.

### Potential Interactions and Implications for Policy

We will have limited direct pharmacokinetic data on interactions between hydroxyurea and traditional polyherbal formulations. Given this uncertainty, our trial includes frequent laboratory safety monitoring and DSMB oversight to identify any signal of interaction or toxicity. If the combined regimen demonstrates efficacy with acceptable safety, the data may contribute to evidence considered by national guideline panels and, potentially, World Health Organization (WHO) working groups on integrative approaches in regions of high SCD prevalence. We recommend that confirmatory trials include dedicated pharmacokinetic/pharmacodynamic substudies to formally evaluate interactions.

### Limitations

This protocol also has a few limitations, such as the open-label design of the study. However, end point assessments will be blinded to mitigate observer bias, following the PROBE design framework, as this study design is considered as good as a double-blinded RCT [53].

### Conclusions

This protocol outlines a scientifically rigorous clinical trial integrating an Ayurveda therapeutic regimen with conventional SCD management. By employing robust methodologies and stringent ethical standards, this study aims to generate high-quality evidence on the safety and efficacy of Ayurveda interventions as an adjunct in SCD management. The findings will contribute valuable insights into the role of integrative medicine in the long-term management of SCD, potentially influencing future therapeutic guidelines and improving patient outcomes.

## Supplementary material

10.2196/79603 Checklist 1 SPIRIT checklist.

## Abbreviations

ADR: adverse drug reaction
AE: adverse event
AIIMS: All India Institute of Medical Sciences
BPI: Brief Pain Inventory
CBC: complete blood count
CONSORT: Consolidated Standards of Reporting Trials
CRF: case record form
CRP: C-reactive protein
DSMB: Data and Safety Monitoring Board
e-CRF: electronic CRF
ELISA: enzyme-linked immunosorbent assay
HbA: adult hemoglobin
HbF: fetal hemoglobin
HbS: hemoglobin S
HRQoL: health-related quality of life
IL-6: interleukin-6
IL-8: interleukin-8
KFT: kidney function test
LDH: lactate dehydrogenase
LFT: liver function test
MFIS: Modified Fatigue Impact Scale
mITT: modified intention-to-treat
NABL: National Accreditation Board for Testing and Calibration Laboratories
PP: per-protocol
PROBE: Prospective, Randomized, Open-Label, Blinded End Point
RCT: randomized controlled trial
SCD: sickle cell disease
SF-8: Short Form-8 Health Survey
SPIRIT: Standard Protocol Items: Recommendations for Interventional Trials
TGF-β: transforming growth factor-β
TM: traditional medicine
VOC: vaso-occlusive crisis
WHO: World Health Organization
